# Autoimmune glial fibrillary acidic protein astrocytopathy in children: a retrospective study

**DOI:** 10.1186/s40001-022-00641-y

**Published:** 2022-01-22

**Authors:** Xiamei Zhuang, Ke Jin, Xiaoming Li, Junwei Li

**Affiliations:** Department of Radiology, Hunan Children’s Hospital, 86 Ziyuan Road, Yuhua District, Changsha, China

**Keywords:** Glial fibrillary acidic protein, Children, Magnetic resonance imaging, Cerebral spinal fluid examination, Virchow–Robin space

## Abstract

**Objective:**

To describe the clinical features of autoimmune glial fibrillary acidic protein (GFAP) astrocytopathy in children.

**Method:**

Data from 11 pediatric patients with autoimmune GFAP astrocytopathy were retrospectively analyzed.

**Results:**

All of the patients showed encephalitis and meningoencephalitis or meningoencephalomyelitis with or without myelitis. 45.4% of the patients had fever, 27.3% headaches, 18.2% dizziness, 18.2% drowsiness, and 18.2% mental disorders. Cerebrospinal fluid (CSF) was detected in all patients. The white blood cell counts (WBC) (90.9%), lactic dehydrogenase levels (72.7%), protein level (36.4%), and adenosine deaminase activity (ADA) level (27.3%) were elevated, and the CSF glucose levels (72.7%) were slightly reduced. Nine patients (90%) were found to have brain abnormalities, of which five (50.0%) patients had abnormal symmetrical laminar patterns or line patterns hyperintensity lesions on T2-weighted and fluid-attenuated inversion recovery (FLAIR) images in the basal ganglia, hypothalamus, subcortical white matter and periventricular white matter. The linear radial enhancement pattern of the cerebral white matter was only seen in two patients, with the most common being abnormal enhancement of leptomeninges (50%). Five patients had longitudinally extensive spinal cord lesions.

**Conclusion:**

The findings of pediatric patients with autoimmune GFAP astrocytopathy are different from previous reports.

## Introduction

In 2016, autoimmune glial fibrillary acidic protein (GFAP) astrocytopathy was first reported as a kind of meningoencephalomyelitis associated with GFAP-IgG, a spectrum of autoimmune inflammatory central nervous system (CNS) disorders, and was subsequently confirmed internationally [1]. This disorder involves meningeal, brain parenchymal, spinal cord, or optic nerve inflammation and injury, and is characterized by corticosteroid response. The main clinical manifestations include fever, headaches, abnormal vision, mental disorders, ataxia, dyskinesia and autonomic dysfunction. It is often accompanied by a hallmark brain linear perivascular radial gadolinium enhancement on magnetic resonance imaging (MRI) [2–5]. Autoimmune GFAP astrocytopathy has been described almost exclusively in adult patients, with the few pediatric cases being reported predominantly as case reports [6–10]. Limited previous studies described the clinical data and MRI characteristics in children as similar to those in adults [10]; however, the clinical features of autoimmune GFAP astrocytopathy in children still need to be defined. In this study, we recruited 11 pediatric cases with autoimmune GFAP astrocytopathy, and focused on the clinical data and MRI features, to identify characteristic features which can increase diagnostic success.

## Patients and methodology

This study protocol was approved by the medical ethics committee of the Hunan Children’s hospital of the University of South China. This retrospective study was approved by the institutional review board. Informed consent was waived due to the retrospective analysis of anonymized data.

### Patients

We enrolled 11 pediatric patients who were admitted to our hospital (Department of Neurology) between January 2018 and April 2021 with encephalitis and meningoencephalitis or meningoencephalomyelitis who only responded to corticosteroid treatment. All of the patients underwent serum and/or CSF testing, and all of them tested positive for GFPA antibodies by cell-based assay (CBA). All of the samples were collected during the early active disease stage, and before corticosteroid treatment. The patients were subjected to detailed clinical examinations, routine cerebrospinal fluid (CSF) examinations, MRI and/or CT analysis. Patient data were retrospectively evaluated through medical record reviews.

### Cell-based assay

At present, the detection methods of GFAP antibodies include histological assay, cell-based assay (CBA) and immunohistochemical assay. This study used the CBA method, as it has high specificity and sensitivity. Cell-based assay (CBA) is an indirect immunofluorescence assay based on cell transfection. The principle of this assay is to transfect GFAP genes into mammalian cells to express GFAP antigens in mammalian cytoplasm. Green fluorescent proteins (GFP) were also expressed in the transfection as an internal reference for detection. Then the transfected cells were fixed onto 96-well microplates to make antigen plates. The GFAP antibodies in human serum, plasma and cerebrospinal fluid samples were detected semi-quantitatively by indirect immunofluorescence.

The autoantibodies were detected by the CBA method. A brief summary of the methodology is as follows: first, the serum to be tested was diluted by 1:10, or the original fluid of cerebrospinal fluid was used to incubate the patch of cells at room temperature for 60 min; then the patch of cells was with detergent 3 times, for 5 min per time. Next, the FITC-labeled goat anti-human IgG secondary antibody was diluted 1:50, and used to incubate the cell patch at room temperature for 30 min. The patch of cells was then cleaned again, sealed, and observed under a fluorescence microscope. The fluorescence signal was significantly higher than the background signal, so the serum located in the cell membrane could be identified as the positive serum of autoantibodies. When the titre of the positive serum was tested, the serum or cerebrospinal fluid was diluted in a gradient, and carried out according to the above method. The titre value of the positive serum was taken as the highest dilution multiple of the positive signal that could be detected.

### Testing of other antibodies

We also tested the patients with GFPA antibodies for some common antibodies, such as myelin oligodendrocyte glycoprotein (MOG), aquaporin-4 (AQP4)-M1, AQP4-M2, anti-N-methyl-D-aspartate receptor (NMDAR) and Yo.

## Results

### Antibody test results

Among the 11 patients who underwent serum and/or CSF testing, we found nine cases of autoantibodies against GFAP in both the CSF and serum samples, and two cases showed autoantibodies against GFAP only in the CSF samples.

Among these 11 patients, two had other common antibodies; one patient was positive for MOG antibodies, and the other was positive for NMDAR antibodies.

### Demographic data and clinical manifestations

There were 5 female patients and 6 male patients (ratio=0.83). The median age at the onset of disease was 52 months (range: 11 months to 128 months). For all patients, we performed a whole-body MR, computed tomography (CT) or ultrasound for tumor screening. Only three patients were found to have tumors (one ependymoma in the brain; one a yolk sac tumor in the testis; and one is uncertain in the retroperitoneal space). More than half of the patients presented with flu-like symptoms. The main symptoms observed included fever (45.4%), headaches (27.3%), dizziness (18.2%), drowsiness (18.2%), and mental disorders (18.2%).

### Cerebrospinal fluid (CSF) findings

Routine CSF analysis included the total white cell counts (WBC), chlorine levels, glucose levels, protein levels, lactic dehydrogenase levels and the adenosine deaminase activity (ADA) levels. CSF abnormalities were found in 10 patients (90.9%), which we have summarized in Table 1. On admission, 10 patients had elevated white blood cell counts (median: 240 $\times$ 10^6^, range: 26–550 $\times$ 10^6^, normal ranges: 0–20 $\times$ 10^6^), and eight patients had slightly elevated lactic dehydrogenase levels (median: 39 IU/L, range: 33–80 IU/L, normal ranges: 0–30 IU/L). Four patients had elevated protein levels (median: 0.75 g/L, range: 0.63–1.56 g/L. normal ranges: 0–0.5 g/L), and three patients had elevated ADA levels (median: 4.5 U/L, range: 4.4–5.2 U/L, normal ranges: 0–4 U/L). The CSF glucose level was reduced in eight patients (median: 2.46 mmol/L, range: 1.83–2.74 mmol/L, normal ranges: 2.8–4.2 mmol/L).

**Table 1:** Data of 11 pediatric patients with autoimmune GFAP astrocytopathy

No.	Gender	Age (month)	Symptoms	Another antibody	CSF abnormality	Main MRI features
1	F	42	Fever	Negative	WBC: 341 $\times$ 10^6^/L glucose: 2.7 mmol/L ADA: 4.48 U/L	Brain: Bilateral juxtacortical WM, cerebellum Meningeal abnormality Sc: normality
2	M	84	Dizzy Emesis	NMDAR	WBC:50 $\times$ 10^6^/L glucose: 2.54 mmol/L lactic dehydrogenase: 33.00 IU/L ADA: 4.40 U/L	Brain: Bilateral basal ganglia, thalamus, periventricular WM and callosal Meningeal abnormality Sc: NA
3	F	11	Mental disorder	Negative	WBC: 120 $\times$ 10^6^/L	Brain: normality Sc: NA
4	M	67	Headache Fever	Negative	WBC: 30 $\times$ 10^6^/L lactic dehydrogenase: 71.00 IU/L ADA:4.5U/L	Brain: NA Sc: NA
5	F	89	Drowsiness dizzy	Negative	WBC: 240 $\times$ 10^6^/L glucose: 2.41 mmol/L lactic dehydrogenase: 80.00 IU/L protein: 1.56 g/L	Brain: Bilateral basal ganglia, thalamus, juxtacortical WM and cerebellum Meningeal abnormality Sc: T1-L1
6	F	52	Fever Cough	Negative	WBC: 55 $\times$ 10^6^/L glucose: 2.46 mmol/L lactic dehydrogenase: 39.00 IU/L protein: 0.88 g/L	Brain: Bilateral basal ganglia, thalamus, periventricular WM and pons Sc: normality
7	M	128	Headache Emesis	Negative	WBC: 550 $\times$ 10^6^/L glucose: 2.54 mmol/L lactic dehydrogenase: 35.00 IU/L ADA: 5.20 U/L	Brain: pons Sc: NA
8	M	14	Mental disorder	Negative	normality	Brain: Bilateral periventricular WM Sc: NA
9	F	68	Drowsiness headache	MOG	WBC: 26 $\times$ 10^6^/L glucose: 2.42 mmol/L	Brain: Unilateral thalamus, cortical gray, juxtacortical WM, deep gray matter and cerebellum Meningeal abnormality Sc: C2-C7, T8-T12
10	M	45	Fever	Negative	WBC: 400 $\times$ 10^6^/L glucose: 2.74 mmol/L lactic dehydrogenase: 36.00 IU/L protein: 0.63 g/L	Brain: Bilateral thalamus, basal ganglia, juxtacortical WM, periventricular WM, cerebellum T1-weighted “radial enhancing” Meningeal abnormality Sc: normality
11	M	24	Fever	Negative	WBC: 55 $\times$ 10^6^/L glucose: 1.83 mmol/L lactic dehydrogenase: 73.00 IU/L	Brain: Bilateral basal ganglia, thalamus, juxtacortical WM, periventricular WM, callosal and pons T1-weighted “radial enhancing” Meningeal abnormality Sc: normality

*ADA* adenosine deaminase activity, *CSF* cerebrospinal fluid, *F* female, *GFAP* glial fibrillary acidic protein, *M* male, *MOG* myelin oligodendrocyte glycoprotein, *MRI* magnetic resonance imaging, *NA* no application, *NMDAR* *N*-methyl-d-aspartate receptor, *Sc* spinal cord, *WM* white matter, *WBC* white blood cell

### MRI findings

**Fig. 1:**
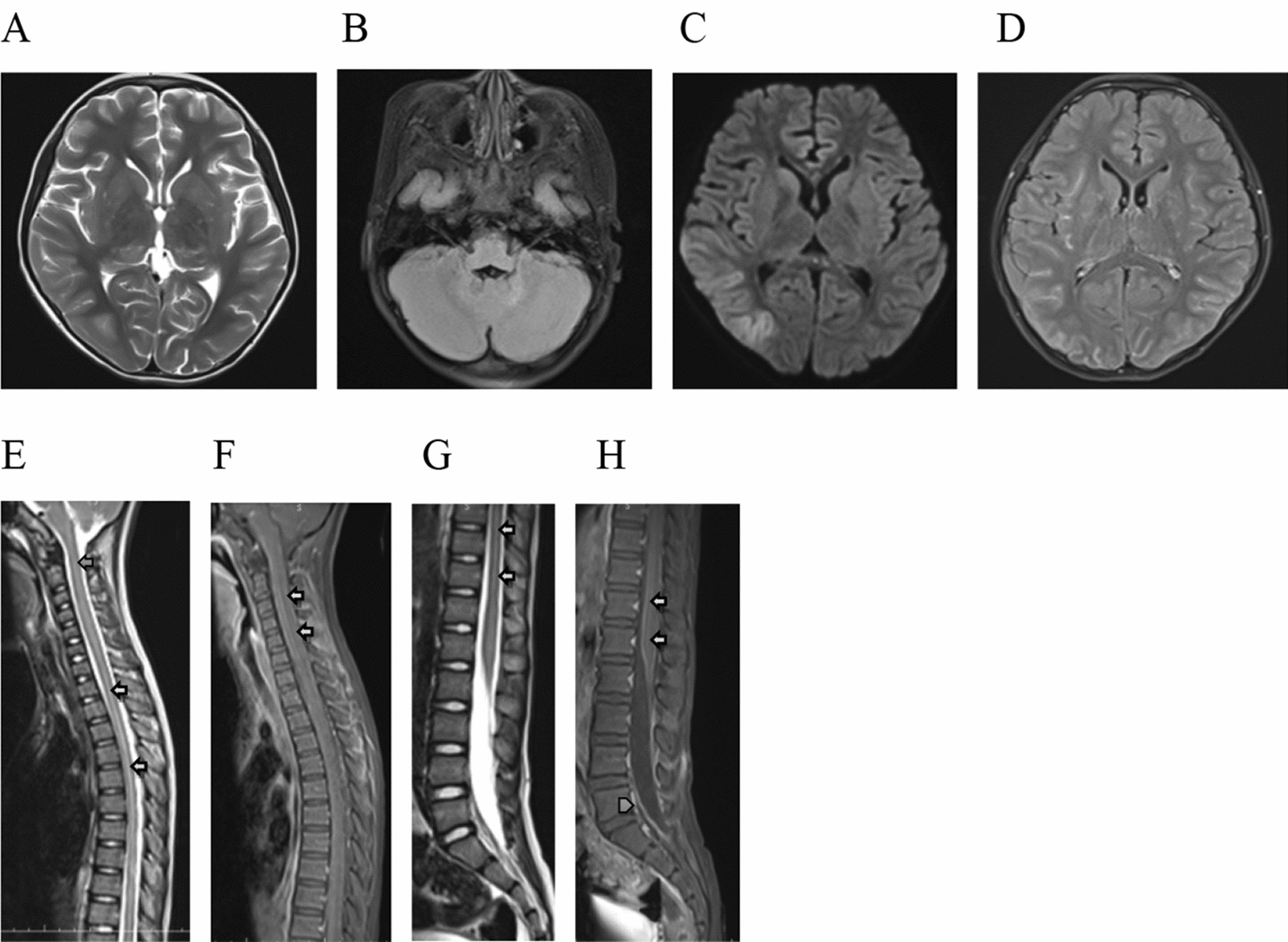
Imaging findings in pediatric patients with autoimmune GFAP astrocytopathy. Magnetic resonance imaging (MRI) showing abnormal hyperintensity lesions on T2-weighted and fluid-attenuated inversion recovery (FLAIR) images in basal ganglia, hypothalamus (**A**) and cerebellum (**B**). Diffusion-weighted sequences imaging (DWI) showed abnormal hyperintensity lesions in the callosum and cortical (**C**). Gadolinium-enhanced brain MRI (T2-FLAIR) showed enhancement leptomeninges (**D**). Spinal cord MRI showed longitudinally extensive spinal cord lesions (LESCLs) (arrows) (**E**, **G**) and slight enhancement (arrows), pia enhancement (arrowhead) (**F**, **H**)

**Fig. 2:**
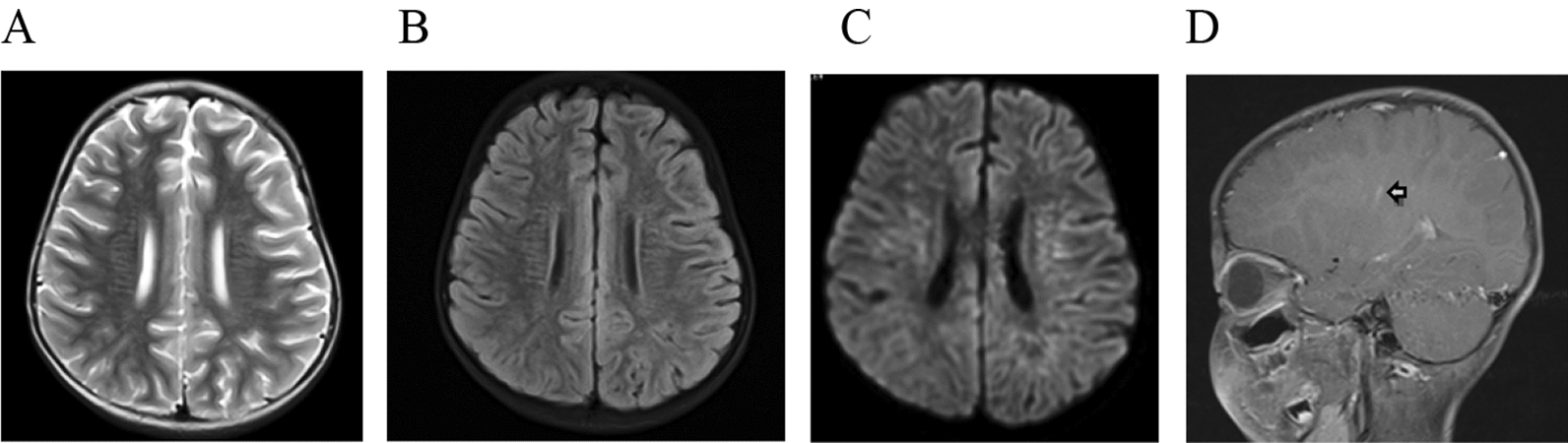
Brain MRI of pediatric patients with autoimmune GFAP astrocytopathy. The abnormal hyperintensity lesions on T2-weighted and FLAIR images were observed in subcortical/around the ventricle white matter (**A**, **B**), DWI showed hyperintensity lesions (**D**). Gadolinium-enhanced brain MRI (T2-FLAIR) showed a linear perivascular radial enhancement pattern (arrow) (**D**)

10 out 11 patients had brain MRI scans at the initial onset of illness, with nine of the of the 10 (90.0%) showing signs of abnormalities after MRI scans. One patient had a brain CT scan, which showed normal results. From the brain MRI, nine patients were shown to have abnormal hyperintensity lesions on T2-weighted and fluid-attenuated inversion recovery (FLAIR) images, which we have summarized in Table 1. The range of abnormal hyperintensity MRI lesions in the thalamus was 60.0%, basal ganglia 50.0%, periventricular white matter 50.0%, juxtacortical white matter 50.0%, cerebellum 40.0%, pons 30.0%, and cortical gray matter 10.0%. In our case, the brain MRI images of five patients show abnormal T2-weighted imaging and FLAIR imaging of hyperintensity signals in the bilateral thalamus, basal ganglia, periventricular white matter and juxtacortical white matter, with symmetrical laminar or line patterns, growing along the vascular space (Virchow–Robin space) (Figs. 1, 2). Lesions with T2-weighted, large, patchy patterns were found, and some lesions also contained cortical, white matter and deep nerve-corpuscles. Cortical abnormalities were found in only one patient. All of the patients received gadolinium enhancement on the brain, of which eight showed abnormal enhancement, with abnormal leptomeninges enhancement being the most common (40.0%) (Fig. 1). In this study, we only found two patients who revealed linear perivascular radial gadolinium enhancement in the white matter, perpendicular to the ventricle (Fig. 2). Other enhancements sometimes appeared to punctuate the basal ganglia and hypothalamus, while most of the lesions showed no enhancement.

Six patients had cervical, thoracic and lumbar spinal cord MRI scans, and while two of these exhibited abnormal imaging, all of them revealed longitudinally extensive spinal cord lesions (LESCLs) (Table 1, Fig. 1). The lesions in the T2-weighted images showed patch and punctate patterns in the spinal cord’s central canal, with two patients showing a slight enhancement.

**Fig. 3:**
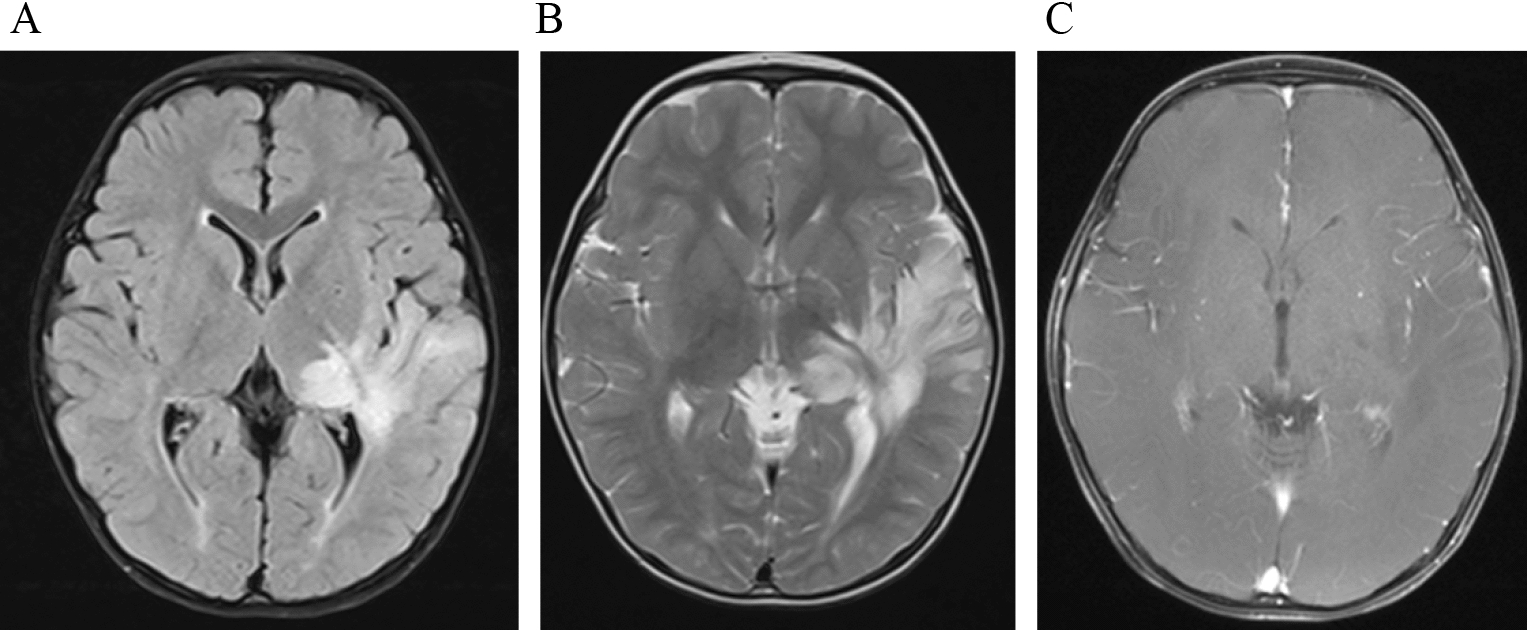
Imaging findings of pediatric patients with autoimmune GFAP astrocytopathy and with positive MOG antibodies. The abnormal hyperintensity lesions on T2-weighted (**A**) and FLAIR images (**B**) were observed in unilateral basal ganglia, hypothalamus and temporal lobe and without abnormal enhancement

Two of the patients had overlapping syndrome. One of these patients had NMDAR antibodies, and this patient’s MRI showed abnormal hyperintension in the bilateral hypothalamus, basal ganglia, periventricular white matter and juxtacortical white matter on T2-weighted imaging and FLAIR imaging with abnormal leptomeninges enhancement. In the second patient, who had MOG antibodies, MRI showed unilateral basal ganglia, hypothalamus and temporal lobe with multiple patches T2WI hypersignal with abnormal leptomeninges enhancement and longitudinally extensive spinal cord lesions (Fig. 3). There was no abnormal enhancement in brain parenchymal lesions.

### Treatment

All of the patients were initially treated with intravenous immunoglobulin (IVIG, 2 g/day for 5 days), eight patients received methylprednisolone (20 mg/kg/day, for 3–5 days). During the follow-up, subsequent oral prednisone (10–60 mg/day) was prescribed for 10 patients. After discharge, one patient was lost to the follow-up.

## Discussion

GFAP is an intermediate astrocyte protein, located between the smaller microfilaments and larger microtubules, and is the primary component of intermediate filaments in astrocytes. It has important biological functions, including the maintenance of astrocytes’ morphological stability, involvement in the blood–brain barrier formation, and the regulation of synapse function. Autoimmune GFAP astrocytopathy is a recently defined autoimmune disease. Since GFAP antibodies have specific biomarkers [4, 11, 12], autoimmune GFAP astrocytopathy diagnosis primarily depends on GFAP antibody detection in the serum or SCF. So far, very few pediatric cases have been reported. In this study, we recruited 11 pediatric patients with GFAP antibodies in GSF and/or serum samples (titre ≥ 1:32) and all of them only responded to corticosteroid treatment. We retrospectively demonstrated the characteristic clinical features of those pediatric patients.

The peak age of onset among our patients was 52 months, and most of them were preschool children. The youngest patient was 11 months, which was younger than any previous study [10, 15]. As far as we know, 11 months is the youngest aged case to have been reported. The female to male ratio was 0.83, which is unlike the ratio found in adult patients. Like the previous studies, most patients had an acute or subacute onset. Clinical manifestations were encephalitis and meningoencephalitis, including fever (45.4%), headaches (27.3%), dizziness (18.2%), drowsiness (18.2%), and mental disorders (18.2%) [3, 9, 10, 13–17]. In our case, all of the patients who suffered from myelitis were shown to have longitudinally extensive transverse myelitis, like in the previous study. Some clinical manifestations seen in adults, such as consciousness disturbance, area postrema syndrome, prolonged gastrointestinal symptoms and so on [7, 18, 19], were rarely seen in pediatric patients, and were not seen in this study. Flanagan reported that 66% of the tumors were detected within 2 years of onset of symptoms, including ovarian teratoma, adenocarcinoma and glioma, yet we found that only three patients had a tumor, which is a much lower frequency than the ones found in other reports [4]^.^ Therefore, patients should be monitored for underlying neoplasms within 2 years of GFAP disease onset.

CSF analysis from our patients with autoimmune GFAP astrocytopathy showed that more than half had characteristic inflammatory CSF. The high frequency of elevated CSF WBC count and slightly elevated protein levels and ADA levels is consistent with the literature [13]. This high frequency of elevated CSF WBC count and protein level is not the only marker of this condition, but also occurs in infectious and neoplastic causes of meningoencephalitis. ADA plays an important role in the growth and differentiation of lymphocytes and macrophages, and some research suggest that the high ADA levels might be associated with immunological pathology during the early stage of autoimmune GFAP astrocytopathy [3]. We found that some patients (72.7%) had a transiently mild decline of glucose levels, while lactic dehydrogenase (72.7%) levels were elevated, which was seldom reported in previous studies and uncommon in immune diseases. Hypoglycorrhachia is normally seen in patients with tuberculous meningitis (TBM). It is caused by release of glycolytic enzymes in the brain and glucose consumption by itself, but the reason for the hypoglycorrhachia in autoimmune GFAP astrocytopathy is unknown. As we know, this is the first time the changes of CSF lactic dehydrogenase in autoimmune GFAP astrocytopathy have been discussed. The lactic dehydrogenase is mainly related to the degree of cell necrosis and the damage of the cell membrane. Elevated lactic dehydrogenase was mainly found in local hypoxic necrosis, bacterial meningitis, cerebral infarction, lymphoma, brain trauma, hydrocephalus and so on. The reasons for the elevation of lactic dehydrogenase in this disease is uncertain, but we suggest that it may be related to the impaired brain cells and injured cell membranes found in autoimmune GFAP astrocytopathy.

From the brain MRI results, 90.0% of patients had abnormalities on T2-weighted and FLAIR sequences. Lesions can involve all of the nervous system, including the cortex, subcortical white matter, nerve nuclei of the deep brain (basal ganglia, hypothalamus, cerebellar dentate nuclei), brainstem, cerebellum, meninges, ventricle and callosum. In our case, the most common abnormalities were laminar patterns or line patterns hyperintense in bilateral thalamus, basal ganglia, periventricular white matter and juxtacortical white matter on T2-weighted imaging and FLAIR imaging. Those lesions, especially in the periventricular white matter and juxtacortical white matter, were just like the distribution along the Virchow–Robin space, which were different from the adult abnormalities [2–5, 10, 12]. The Virchow–Robin space surrounds the walls of vessels as they course from the subarachnoid space through the brain parenchyma, and it does not communicate directly with the subarachnoid space. The Virchow–Robin space can provide the changes for the extraneous antigen into the brain, and the interstitial fluids partly participate in the immunomodulatory effect. Also, it can be one way in which the disease spreads [28]. The autoimmune GFAP astrocytopathy revealed marked inflammatory responses around the perivascular region and small blood vessels [2, 5, 13, 20, 21], emanating from GFAP-enriched peri-lateral ventricular regions, frequently seen in the basal ganglia, hypothalamus, and the white matter (subcortical or/and around the ventricle). We speculated that in our case, the five patients who showed bilateral hyperintense in the thalamus, basal ganglia, periventricular white matter and juxtacortical white matter, were related to the accumulation of inflammatory cells, antigens and antibodies in the perivascular space and the Virchow–Robin space. It should be noted that previous studies have proposed that bilateral thalamus abnormal hyperintense signal was the characteristic manifestation of autoimmune GFAP astrocytopathy [3], and some studies can see similar signal changes in the bilateral basal ganglia, thalamus and white matter [2–5, 10, 12]. The characteristic pattern of brain linear perivascular radial gadolinium enhancement in the white matter, perpendicular to the ventricle, was only seen in two patients in our study, which is also consistent in the pattern of inflammation around the small vessels [22]. However, in our cases, it is markedly lower than in adults’ patients. Although previous studies have pointed out that brain linear perivascular radial gadolinium enhancement is a characteristic manifestation of the disease, Jonathan Wickel also pointed out that radial perivascular emphasis is not necessarily associated with GFAP antibodies [29].

Four patients showed abnormal enhancement of leptomeninges, which rarely occurs in autoimmune encephalitis. GFAP, an intermediate astrocyte protein between the smaller microfilaments and larger microtubules, is the primary component of intermediate filaments in astrocytes. It has important biological functions, including the maintenance of astrocytes’ morphological stability, involvement in blood–brain barrier formation, and the regulation of synapse function. Perhaps that enhancement was caused by gadolinium leaking from the damaged blood–brain barrier, but the reason is unknown. Previous studies indicated that the radial enhanced patterns were relative to the gadolinium leakage from compromised vascular walls [2, 23]. This raises some questions: Why are radial enhancing patterns rare in pediatric patients? Is it that pediatric patients of this disease are seldom injured in the small vascular walls? Previous studies have shown that all of them were normal on DWI, but our findings were different. The reason for this is also uncertain. Myelitis in pediatric patients is relative rare compared adult patients [10, 14, 24–27]. In our case, the two patients with myelitis showed longitudinally extensive myelitic abnormalities, and a slight enhancement.

## Conclusion

In conclusion, clinical manifestations were encephalitis and meningoencephalitis. Brain MRI imaging revealed hyperintensity lesions in the bilateral thalamus, basal ganglia, periventricular white matter and juxtacortical white matter, with symmetrical laminar or line patterns, especially with the abnormal enhancement of leptomeninges, with characteristic inflammatory CSF, which suggested the possibility of autoimmune GFAP encephalitis. Our data suggest that this disease was corticosteroid-responsive only. Therefore, early diagnosis and treatment is very important. CSF and serum GFAP-IgG should be examined in pediatric patients with the above as early as possible.

This study provides some interesting findings and issues that are important for clinical diagnosis. However, we only had 11 pediatric patients with autoimmune GFAP astrocytopathy, which represents a very small sample size, so future studies should be undertaken in a larger population.

## Data Availability

All data generated or analysed during this study are included in this published article.

## Abbreviations

ADA: Adenosine deaminase activity
CBA: Cell-based assay
CNS: Central nervous system
CSF: Cerebrospinal fluid
CT: Computed tomography
DWI: Diffusion-weighted sequence imaging
FLAIR: Fluid-attenuated inversion recovery
GFAP: Glial fibrillary acidic protein
LESCLs: Longitudinally extensive spinal cord lesions
MRI: Magnetic resonance imaging
TBM: Tuberculous meningitis
WBC: White cell counts
